# Predictive value of traction force measurement in vacuum extraction: Development of a multivariate prognostic model

**DOI:** 10.1371/journal.pone.0171938

**Published:** 2017-03-03

**Authors:** Kristina Pettersson, Khurram Yousaf, Jonas Ranstam, Magnus Westgren, Gunilla Ajne

**Affiliations:** 1 Obstetrics and gynecology, Karolinska University Hospital, Stockholm, Sweden; 2 Clintec, Karolinska Institute, Stockholm, Sweden; 3 School of Technology and Health, Royal Institute of Technology, Stockholm, Sweden; 4 Statistician, Swedish Patient Insurance, Stockholm, Sweden; Wayne State University, UNITED STATES

## Abstract

**Objective:**

To enable early prediction of strong traction force vacuum extraction.

**Design:**

Observational cohort.

**Setting:**

Karolinska University Hospital delivery ward, tertiary unit.

**Population and sample size:**

Term mid and low metal cup vacuum extraction deliveries June 2012—February 2015, n = 277.

**Methods:**

Traction forces during vacuum extraction were collected prospectively using an intelligent handle. Levels of traction force were analysed pairwise by subjective category strong versus non-strong extraction, in order to define an objective predictive value for strong extraction.

**Statistical analysis:**

A logistic regression model based on the shrinkage and selection method lasso was used to identify the predictive capacity of the different traction force variables.

**Predictors:**

Total (time force integral, Newton minutes) and peak traction (Newton) force in the first to third pull; difference in traction force between the second and first pull, as well as the third and first pull respectively. Accumulated traction force at the second and third pull.

**Outcome:**

Subjectively categorized extraction as strong versus non-strong.

**Results:**

The prevalence of strong extraction was 26%. Prediction including the first and second pull: AUC 0,85 (CI 0,80–0,90); specificity 0,76; sensitivity 0,87; PPV 0,56; NPV 0,94. Prediction including the first to third pull: AUC 0,86 (CI 0,80–0,91); specificity 0,87; sensitivity 0,70; PPV 0,65; NPV 0,89.

**Conclusion:**

Traction force measurement during vacuum extraction can help exclude strong category extraction from the second pull. From the third pull, two-thirds of strong extractions can be predicted.

## Introduction

The impact of traction force on perinatal outcome after vacuum extraction is insufficiently studied. Although vacuum extraction is a common alternative to second stage caesarean section (CS), the method has not developed substantially since its clinical implementation about 50 years ago, and the American College of Obstetricians and Gynecologists regret that “few specific aspects of (…) technique have been studied”[1]. The rate of operative vaginal delivery varies widely in Europe, with a median of 7,5% (0,5–16,4) in 2010 [2]. Recently, several authors have expressed concern about increasing CS rates [1, 3] and in the US decreasing use of vacuum extraction [4] due to fear of severe neonatal complications. One possible response to this tendency is action to increase safety in vacuum extraction by investigating specific risk factors, and in this project we have chosen to focus on traction force and awareness.

In current practice, the obstetrician bases his or her judgment of extraction progression solely on the subjective impression of”progressive descent with moderate traction” [5]. However, there is no established definition of this moderate traction, neither in clinical practice nor in the literature, and observed levels of force vary between studies. Two studies on plastic cup extractions suggest that the majority of deliveries require no more than the force equivalent to 11,5 kg (approximately 112 Newton)[6, 7]. Our research group found a different result in an observational study of 200 metal cup extractions, with average peak traction forces of 176, 225 and 241 N, depending on subjective category as easy, average or strong extraction respectively[8].

Our aim was to develop a predictive model for early recognition of strong traction force category based on objective measurements of exerted traction force, thereby facilitating the decision to terminate a difficult attempt.

## Methods

Traction force data was collected from 277 vacuum extraction deliveries at the delivery ward, Karolinska University Hospital from June 2012 to February 2015. The department is a tertiary unit, with nearly 5000 deliveries every year. All term, singleton, low and mid cavity metal cup extractions were eligible, including aborted attempts followed by CS or, on rare occasions, by forceps. A total of 855 vacuum extraction deliveries were performed during this period. Of these, approximately 60% were low or mid high vacuum extractions, and 40% outlet. The total number of vacuum extractions was 8% of all deliveries at the hospital, which is similar to the Swedish national rate. [9]

As a result of risk group identification, we excluded outlet extractions, since our previous observational study on traction force levels showed no strong extractions within this group[8]. High extractions (vertex above the ischiatic spines) are not common practice in Swedish obstetrics. Plastic cup extractions were excluded by default, since the handle device for force measurement and documentation requires attachment to a metal cup. Obstetricians were recommended to use metal cup for all vacuum extractions during the study period. An MD (first author) collected maternal, intrapartal and neonatal data from the medical charts. Ethical approval was given by the local ethics committee of Stockholm, Sweden (D:nr 2012/1553-31/1, 2016/211-32).

### Technical equipment

To measure the traction force employed during vacuum extraction, we used an intelligent handle attached to the chain of a regular metal cup (Bird 50 mm, 80 kPa) as described in a previous study[8]. The handle contains a load cell, a well established type of force sensor[10]. The intelligent handle also encapsulates the necessary instrumentation (e.g. battery, signal conditioning, processor and Bluetooth transceiver) to enable force measurements from the load cell to be wirelessly transmitted to a computer. The computer in turn, records the force measurements which are then retrieved and utilized to compute traction force variables.

### Validation of the device

The force sensor (load cell) was calibrated using standard force-transfer methods [11](using a pre-calibrated material testing machine (Instron E3000®, Instron, Norwood, MA, US). Regular inspection, calibration and maintenance of the intelligent handle during the course of this study depicted no deviation in its performance and good accuracy of force measurements.

### Traction forces

In every vacuum extraction delivery, the highest momentary peak force (Newton, N) during each individual pull, as well as the total force (Newton minutes, Nmin), area under the curve, during each pull was measured. One pull corresponds to one uterine contraction. The differences in peak and total force between the first and subsequent pulls within each extraction were also calculated. We chose not to include other clinical data as possible predictors when developing our test, since we aimed for simplicity in the clinical situation.

The candidate predictors (i.e. variables included in the statistical analysis) were peak and total force during each individual pull one through three, and the difference in force between the second and first pull, and the third and first pull. Accumulated total force after the second and third pulls.

In the analyses, the outcome was binary: strong or non-strong extraction as subjectively classified by the obstetrician following the procedure. Many countries and clinics use a similar three grade scale for perceived difficulty or required traction effort (e.g. easy, moderate, strong), and we have chosen the term “strong” as it is exemplified in RCOG guidelines [5]. For analyses, we clustered easy and moderate as non-strong. Paired traction force data (strong vs non-strong) were analysed using logistic regression with shrinkage and selection, resulting in two receiver operating curves (ROC): 1–2 is based on traction forces in pull one and two, while 1–3 additionally includes traction forces from the third pull.

Per definition, there was no missing item data regarding traction force measurement, since the included cases were collected from the traction force documentation software. Seven units (cases) were excluded by default because there was no documentation of subjective category: no outcome data available. However, not all eligible cases during the study period underwent extraction with the traction force measuring handle; a total of 546 mid or low extractions were eligible. In 269 of these, the measurement device was not used. The most likely explanations for this unintended exclusion are: technical (equipment not available due to maintenance) or compliance factors (assistant nurses or doctors not comfortable or sufficiently trained to use the equipment).

### Statistical analyses

The binary outcome variable was strong or non-strong extraction category, while the continuous predictor variables included in the analyses were the different traction force data described above.

Descriptive statistics were computed with Statistica, and are presented as mean±SD or median (min–max) as appropriate. The Student’s t-test was used to test mean values of normally distributed data and the Mann–Whitney U-test for data with a skewed distribution. The chi-square test was used for dichotomous data. A value of P < 0.05 was considered statistically significant.

A prediction model was developed using a logistic regression model based on the shrinkage and selection method lasso (Least Absolute Shrinkage and Selection Operator)[12]. The method involves penalizing the absolute size of the coefficients of a regression model, based on the value of a tuning parameter λ. The larger the applied penalty, the further estimates are shrunk towards zero. This makes the coefficient of irrelevant variables zero: an automatic variable selection procedure. Cross validation is used to choose λ and to assess the predictive accuracy of the model, which protects against overfitting. Calculations were carried out using Stata v13.1 [13]and the R-library glmnet[14]

## Results

The prevalence of strong extraction was 26%. Eight cases contributed force data from only one pull, and 83 from two pulls. The remaining 186 cases contributed three or more pulls, including all 70 strong category extractions. Clinical characteristics for included vacuum extraction are shown in Table 1.

**Table 1:** Maternal, obstetrical and fetal characteristics: strong versus non-strong force.

	Subjective category Strong force	Subjective category Non-strong force	p-value
	n = 70	n = 201	
**Age (#)**	31 ± 5	31 ± 5	NS
**BMI (§)**	25 (18–41)	25 (17–43)	NS
**Gestational age, days (#)**	282 ± 14	278 ± 10	p<0.05
**Duration first stage, minutes (§)**	630 (165–1380)	540 (100–1680)	NS
**Duration extraction, minutes (#)**	12 ± 6	8 ± 4	p<0.001
**Number of pulls (#)**	5 ± 2	4 ± 1	NS
**pH umb a (#)**	7.21 ± 0.1	7.19 ± 0.1	NS
**Birth weight, g (#)**	3828 ± 438	3553 ± 469	p<0.001
**Nulliparous, n**	58/70	155/201	NS
**Indication, n:**			
**Susp fetal compromise**	23/69	96/200	p<0.05
**dystocia**	46/69	104/200	p<0.05
**Engagement of fetal head, n**			
**mid**	49/70	79/201	p<0.001
**low**	21/70	122/201	p<0.001
**Position of fetal head, n**			
**OAP**	51/65	182/196	p<0.01
**OPP**	14/65	14/196	p<0.01
**Pop-off (≥1), n**	24/70	10/201	p<0.001
**Asphyxia (pH<7.1), n**	9/68	22/191	NS
**Failed/converted extraction, n**	28/70	1/200	p<0.001
**Shoulder dystocia, n**	4/70	6/200	NS
**Hypoxic ischemic encephalopathy (HIE), n**	4/70	1/200	p<0.01

# mean ± sd.

§ median (min-max).

NS non significance.

n number.

T-test or Mann–Whitney U-test for numerical data. Chi square test for nominal data

Missing data: Seven vacuum extractions were not categorized by the obstetrician.

Traction force variables are shown in Table 2.

**Table 2:** Traction force variables, strong vs non-strong, Newton (N) or Newton minutes (Nm).

	Subjective category Strong	Subjective category Non-strong	p-value
**Pull 1 total (Nm)**	77 (21–261)	56 (1–175)	p<0.001
**Pull 2 total (Nm)**	87 (27–268)	43 (1–173)	p < .0001
**Pull 3 total (Nm)**	87 (14–324)	35 (1–151)	p<0.001
**Pull 1 peak (N)**	195 (89–404)	162 (5–361)	p<0.001
**Pull 2 peak (N)**	199 (76–371)	131 (28–436)	p<0.001
**Pull 3 peak (N)**	195 (91–453)	120 (15–369)	p<0.001
**Pull 1+2 total (Nm)**	193 (51–449)	115 (1–407)	p<0.001
**Pull 1+2+3 total (Nm)**	517 (71–1380)	180 (1–857)	p<0.001
**Diff pull 2–1, total (Nm)**	15 (-75-222)	-16 (-122- 98)	p<0.001
**Diff pull 3–1, total (Nm)**	10 (-67-278)	-26 (-147-95)	p<0.001
**Diff pull 2–1, peak (N)**	2 (-158-172)	-25 (-233-119)	p<0.001
**Diff pull 3–1, peak (N)**	6 (-201-254)	-49 (-198-149)	p<0.001

Median (min-max). Mann-Whitney U test.

The variables selected in the best prediction model for pull 1–2 were: peak force (N) pull one and two; total force (Nmin) pull one; accumulated total force pull one plus two; difference in total force between pull two and one. The best prediction model for pull 1–3 included peak force pull three; total force pull two and three; difference in total force between pull three and one.

Model performance is presented as Area Under the Curve (AUC). For pull1-2 AUC was 0,85 (CI 0,80–0,90) and for pull 1–3 0,86 (CI 0,80–0,91), S1 and S2 Figs. After the second pull, sensitivity was 0,87; specificity 0,76, resulting in a negative predictive value (NPV) of 0,94 and a positive predictive value (PPV) of 0,56. After the third pull, sensitivity was 0,70; specificity 0,87; NPV 0,89 and PPV 0,65.

## Discussion

### Main findings

We found a strong negative predictive value of traction force at second and third pulls (NPV 0,94 and 0,89 respectively) and at the third pull, a capacity to predict nearly two-thirds of strong extractions (PPV 0,65). The variables selected in the best prediction model for pull 1–2 were: peak force (N) first and second pull, total force (Nmin) pull one, accumulated total force pull one plus two, and the difference in total force between the second and first pull. The best prediction model for pull 1–3 included peak force third pull, total force second and third pull, as well as the difference in total force between the third and first pull.

### Strengths and limitations

The Area Under the Curve for the ROC is well over 0,7 in both subtests (0,85 and 0,86 respectively), which could serve as a basic ascertainment of the model´s performance. Prediction at the second pull has the obvious advantage of earlier diagnosis. However, with its high sensitivity and low specificity, this model risks a large number of false positive results, which could be a problem if the obstetrician had no other basis for his or her decision on whether to continue the extraction. It is therefore crucial to emphasize that this test is intended as an additional piece of information, rather than to replace established clinical guidelines.

With a PPV of 0,56 and NPV of 0,94, the prognostic function at the second pull could be characterized as re-assuring if negative test, and increased watchfulness if positive. A positive test during the third pull (PPV of 0,65 and NPV 0,89) predicts nearly two-thirds of strong extractions, and therefore makes a somewhat stronger case for considering interruption of the extraction and conversion to a different mode of delivery.

One way of increasing the PPV of the test might have been to narrow the risk group by clinical specifics known to carry an increased risk of strong extraction, thereby increasing the prevalence of the outcome variable. The test scope would then be limited to for example mid station, occiput posterior position extractions, with duration more than 12 minutes (see Table 1). Again, we wanted to develop a more universal test, since the risk of losing one´s judgement during a difficult procedure probably does not vanish in low-risk situations.

The reliability of measurement of the exposure (traction forces) is optimized by monthly checks for accuracy of the device and software by an engineer. Measurement of the outcome (subjective category) has an inherent reliability issue because of its subjective nature and lack of criteria. However, in an observational study comparing objective traction forces with subjective category of the extraction, the authors found significantly different (increasing) traction forces in easy, average and maximal extractions[15], suggesting a basic inter-rater reliability. In our current study, only one clinician (first author) obtained the outcome data for all cases, but this process involves no interpretation or ambiguity.

Obstetricians were blinded to the exposure when categorizing the extraction, to make sure that the outcome actually measured subjective category and not merely a reflection of the exposure (objectively measured traction force). Not blinding at this level would have introduced a validity problem. As mentioned earlier, we do not consider selection bias by exclusion of outlet extractions to be a problem when setting out to examine high levels of traction force, since these low risk extractions are virtually never classified as strong.

Although there is a relatively large proportion of missing unit data (276/546 eligible extractions), we do not expect this to infer any substantial selection bias, since we have reasonable non-systematic related explanations for the failure to include these cases: The doctor, who might harbor an interest in”hiding” expectedly difficult cases from surveillance, is not the person in charge of the inclusion process; this is done by the assisted nurse. We have continuously interviewed the assistant nurses in order to detect any systematic problems, and this revealed only one doctor declining to use the measuring device. On the other hand, we are aware of examples of technical problems. These range from several week long periods when the technical handle or software was absent due to maintenance, and a long upstart period when initially only a minority of the assistant nurses were trained to use the equipment. The total eligible study population and the included patients have a similar rate of failed extractions, which strengthens the notion that this missing data does not induce bias. In summary, our conclusion is that this information taken together decreases the suspicion of any systematic exclusion.

Even though this study is observational rather than interventional, we are aware that raising attention to a domain can in itself influence behavioral changes and thereby to some extent outcome. This is known as the Hawthorne effect. Certainly, with new technical equipment and objective documentation of individual performance, there is a risk of causing obstetricians to make different choices than they would have before the introduction of a new device. This issue will be further investigated in a coming cohort study from our research group.

Generalizability of these results may be limited by the context of the study; conducted at Karolinska University Hospital delivery ward, with a frequency of 8% vacuum extraction deliveries during the study period, approximately 60% mid or low, and a fourth of operations categorized as strong, the distribution might not be representative in all delivery settings. The rate of vacuum extraction delivery varies in individual Swedish obstetric units from 4,8 to 13,2% [9], and it cannot be excluded that this difference in frequency also entails variations in other aspects of clinical practice in operative vaginal deliveries. Previous studies on traction force [6, 7] with plastic cup extractions have a lesser proportion of mid cavity deliveries and considerably lower levels of average traction force. However, this is not necessarily a problem of generalizability, since it does not rule out the ability of the test to predict high risk extractions outside of the study context, but rather implies that in a clinical circumstance where lower traction force levels are employed, and low cavity or outlet is the predominant fetal head station, such a test may not be of clinical relevance. On the other hand, the uncertainty of clinical assessment of fetal head station is a well-known issue [16] and comparisons between institutions may not be straight forward. In our previous study (8) more than 60 percent of mid cavity extractions succeed without applied strong force, which may support the continued use of vacuum extraction at this fetal head station.

In the development of prediction models, the risk of initial over-fitting is well established [17], and we have addressed this by using the cross-validation built into the lasso regression model[18].

### Interpretation

Prognostic testing increases the objectivity of a clinical decision, leaving less room for poor subjective judgement by the obstetrician, identified as a common factor in a UK confidential enquiry on 36 cases of perinatal mortality in vacuum extraction [19]. We hypothesize that this method leads to increased patient safety by potentially avoiding and reducing the duration of strong extractions. Our focus in this project is perinatal safety rather than maternal outcome, and the following comments reflect this focus.

There are plausible patophysiological models as to how strong traction force might cause adverse effects on the fetal head by tearing at the falx cerebri and cause bleeding from emissary veins[20], and the forces acting during operative vaginal delivery have been described in detail elsewhere[21, 22]. The most severe complications occurring after vacuum extraction are subaponeurotic (subgaleal) hemorrhage, intracranial hemorrhage, and asphyxia related morbidity, such as hypoxic ischemic encephalopathy[23]. The incidence of these rare events varies in different studies from 12-19/10 000 (ICH)[24–26] and 4-15/10 000 (subgaleal)[27–30]. In a large Swedish data base study, authors found that encephalopathy, occurs at a similar rate in non-planned CS and vacuum extractions, 83-97/10 000 respectively. [26]. In our previous study[8], we observed a seven percent prevalence of (mild) hypoxic ischemic encephalopathy in strong category extraction. There is also some data suggesting that hypoxic encephalopathy and subgaleal hematoma might be comorbid conditions[29]. This concept of increased risk of specific complications after strong vacuum extraction warrants further investigation of how to apply this knowledge in clinical practice. Identifying those cases of vacuum extraction with an increased risk might lower the frequency of complications, given the belief that strong traction can cause asphyxia, subgaleal hematoma or intracranial hemorrhage.

## Conclusion

Traction force measurement during vacuum extraction can help exclude strong category extraction from the second pull. From the third pull, two-thirds of strong extractions can be predicted.

A valuable consequence of testing is that less experienced professionals can identify high risk situations that would otherwise require great seniority to detect. Vacuum extraction is often regarded as a simple method to master, as opposed to forceps, and is frequently performed by junior doctors who might benefit from objective prognostic methods.

For external validation of this prediction model, and to test the hypothesis that feed back on imminent strong extraction can decrease neonatal morbidity, we are currently setting up a randomized clinical trial. The interventional arm consists of a vibrating notification signal in the handle, based on the predictive tests described in this article. The coming study is intended to contribute causal information to the overall aim of this project, namely to evaluate traction force as an independent risk factor for perinatal morbidity. We believe that if vacuum extraction could be conducted and taught in a more controlled manner, it might be considered a viable option in mid and low fetal head station situations, even in countries where most of these women are currently delivered by CS.

## Supporting information

S1 Fig ROC pull 1–2. AUC 0.85, Specificity 0.76, Sensitivity 0.87, PPV 0.56, NPV 0.94.
(TIF)

S2 Fig ROC pull 1–3. AUC 0.86, Specificity 0.87, Sensitivity 0.70, PPV 0.65,NPV 0.89.
(TIF)

S1 File Data file. (XLSX)
