# Supplementary figures and images for: Predictive value of traction force measurement in vacuum extraction: Development of a multivariate prognostic model
Source: PLoS One. 2017 Mar 3;12(3):e0171938. doi: 10.1371/journal.pone.0171938 (PMC5336195; doi:10.1371/journal.pone.0171938)

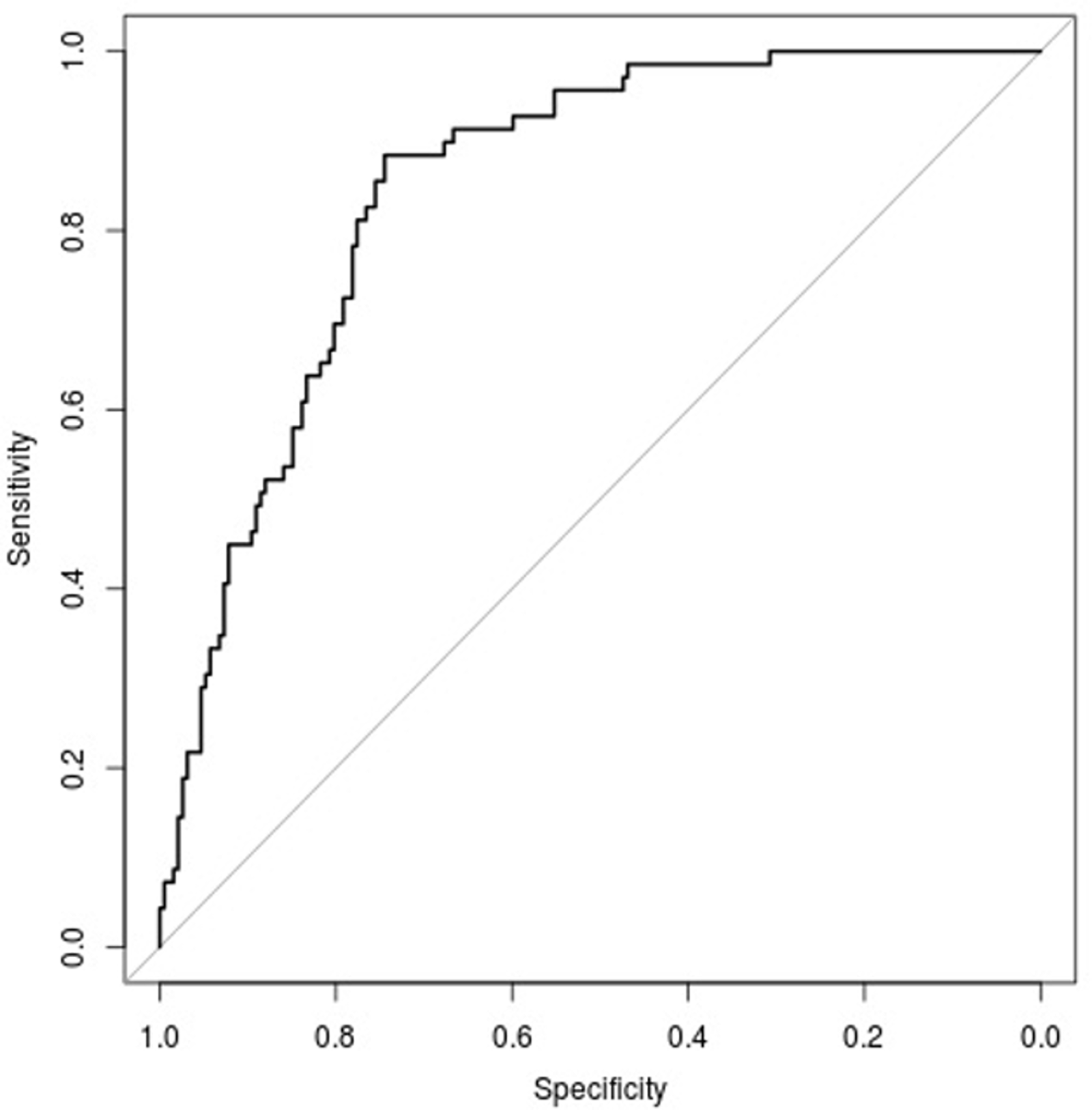

Supplement: S1 Fig — AUC 0.85, Specificity 0.76, Sensitivity 0.87, PPV 0.56, NPV 0.94. (TIF) [file pone.0171938.s001.tif]

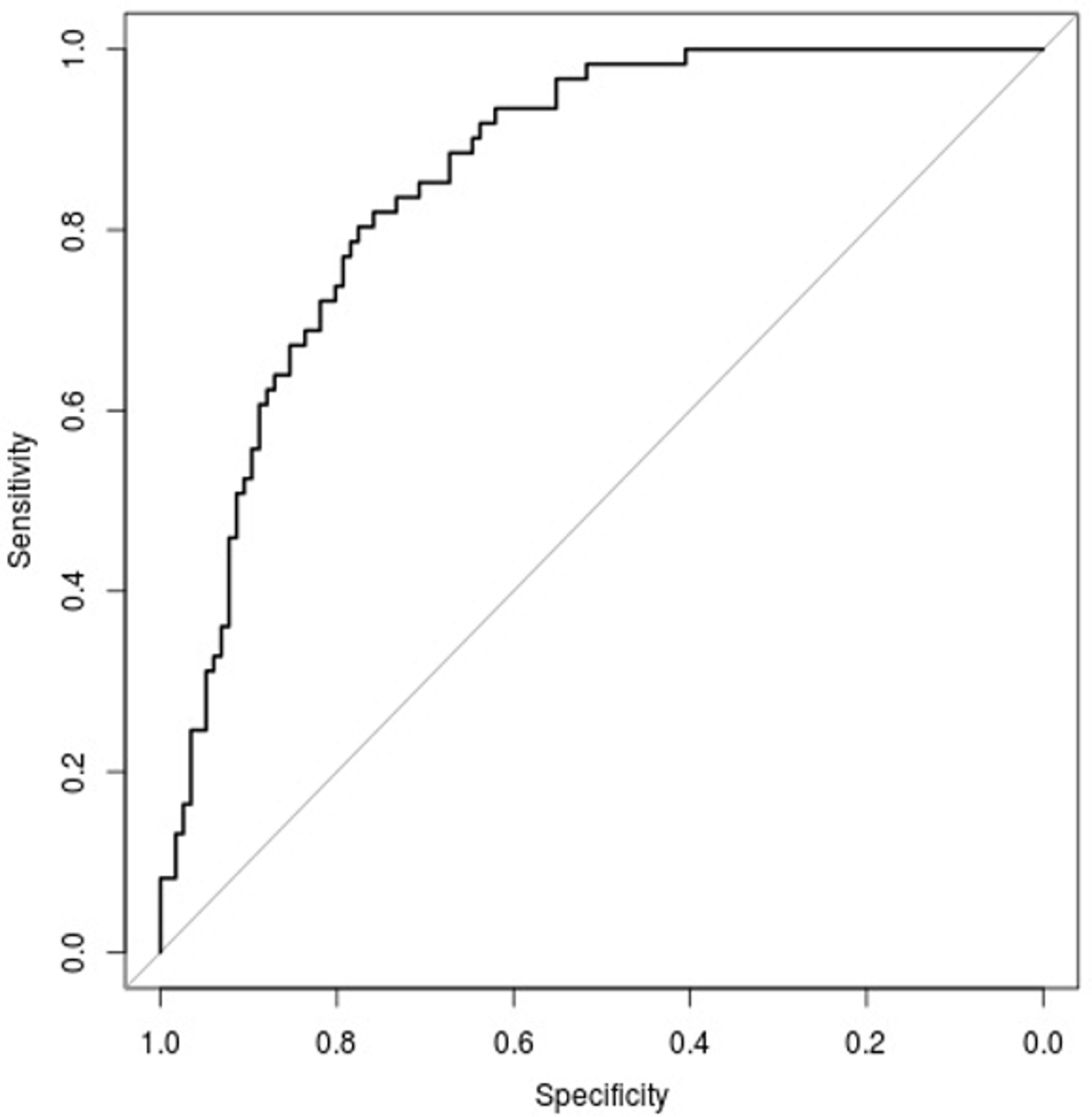

Supplement: S2 Fig — AUC 0.86, Specificity 0.87, Sensitivity 0.70, PPV 0.65,NPV 0.89. (TIF) [file pone.0171938.s002.tif]
